# Microwave-Assisted Alkali Pre-Treatment, Densification and Enzymatic Saccharification of Canola Straw and Oat Hull

**DOI:** 10.3390/bioengineering4020025

**Published:** 2017-03-26

**Authors:** Obiora S. Agu, Lope G. Tabil, Tim Dumonceaux

**Affiliations:** 1 Department of Chemical and Biological Engineering, University of Saskatchewan, 57 Campus Drive, Saskatoon, SK S7N 5A9, Canada; osa542@mail.usask.ca; 2 Agriculture and Agri-Food Canada, Saskatoon Research Centre, 107 Science Place, Saskatoon, SK S7N 0X2, Canada; Tim.Dumonceaux@AGR.GC.CA

**Keywords:** pellet, microwave pre-treatment, biomass, tensile strength, dimensional stability, pellet density, glucose yield

## Abstract

The effects of microwave-assisted alkali pre-treatment on pellets’ characteristics and enzymatic saccharification for bioethanol production using lignocellulosic biomass of canola straw and oat hull were investigated. The ground canola straw and oat hull were immersed in distilled water, sodium hydroxide and potassium hydroxide solutions at two concentrations (0.75% and 1.5% *w*/*v*) and exposed to microwave radiation at power level 713 W and three residence times (6, 12 and 18 min). Bulk and particle densities of ground biomass samples were determined. Alkaline-microwave pre-treated and untreated samples were subjected to single pelleting test in an Instron universal machine, pre-set to a load of 4000 N. The measured parameters, pellet density, tensile strength and dimensional stability were evaluated and the results showed that the microwave-assisted alkali pre-treated pellets had a significantly higher density and tensile strength compared to samples that were untreated or pre-treated by microwave alone. The chemical composition analysis showed that microwave-assisted alkali pre-treatment was able to disrupt and break down the lignocellulosic structure of the samples, creating an area of cellulose accessible to cellulase reactivity. The best enzymatic saccharification results gave a high glucose yield of 110.05 mg/g dry sample for canola straw ground in a 1.6 mm screen hammer mill and pre-treated with 1.5% NaOH for 18 min, and a 99.10 mg/g dry sample for oat hull ground in a 1.6 mm screen hammer mill and pre-treated with 0.75% NaOH for 18 min microwave-assisted alkali pre-treatments. The effects of pre-treatment results were supported by SEM analysis. Overall, it was found that microwave-assisted alkali pre-treatment of canola straw and oat hull at a short residence time enhanced glucose yield.

## 1. Introduction

The world relies on fossil fuels for its energy usage and the sources of these fossil fuels are coal, oil and natural gas. Any event that threatens their availability affects the cost of supply such as is being experienced with petroleum supply [1]. However, the negative impact of fossil fuels on the environment is the increasing problem of greenhouse gas emissions. These emissions have attracted global interest in the search for alternative, non-petroleum-based sources of energy [1,2]. These renewable energy sources include solar energy, biomass, wind, hydroelectric and other sources that are more environmentally friendly [3]. 

According to Alvira et al. [2] and Balat et al. [3], fuel ethanol can be produced from renewable biomass such as sugar, starch or lignocellulosic materials. It is clear that lignocellulosic materials from agricultural residues are interesting alternative and the biomass feedstocks for production of bioethanol has shifted from first-generation feedstocks (grains and oilseeds) to second- (cellulosic biomass from crop residues and dedicated energy crops) and third-generation feedstocks (microalgae) in order to proliferate renewable energy production [4,5].The second- and third-generation feedstocks have been attracting research interest because they are considered as non-food material, have no competition with the food industries, are less expensive than conventional agricultural feedstocks, are available worldwide, are renewable and a good source of raw materials for developing bio-based products and bio-chemicals such as bioethanol or biodiesel [4,6]. Liquid biofuel can replace the fossil fuels used in transportation, electricity, heat and plant generation for domestic and industrial purposes and bioethanol current blend has facilitated positive ethanol–petrol mixtures [7]. In Canada, 5% renewable content in gasoline has been mandated since 2010 along with 2% renewable content in diesel fuel and heating oil since 2011 [5,8]. In the USA, 10% of total gasoline consumption by 2020 have been targeted and the production of 136 billion litres of biofuel is targeted [5].

Lignocellulosic materials include agricultural residues and by-products such as canola straw, wheat straw, rice straw, oats straw, corn stover, corn fibre, oat hull, rice hull, etc. [9]. According to Sanchez and Cardona [10], annual production of lignocellulosic biomass residue was estimated at 1 × 10^10^ Mega gram Mg worldwide. In Canada, the estimated average agricultural residue generated over a 10-year period (2001–2010) was 82.35 million dry Mg/y Saskatchewan recorded the highest at 17.38 million dry Mg/y [11]. These agricultural residues and by-products can be used for conversion into bioethanol.

Canola and oat are major crops grown in Canada. Canola, an oilseed, has an estimated crop production of 15,555.1 million metric tonnes per year (mmt/y) and Saskatchewan’s production is estimated at 8.9 mmt/y. Oat production is estimated to be 2907.5 mmt/y, and Saskatchewan (1.6 mmt/y), Manitoba and Alberta are the major producers in Canada [12].

The pre-treatment of lignocellulose material from agricultural residue is a key step for efficient utilization of biomass for ethanol production. Pre-treatment helps in the breakdown of cell walls and internal tissues of the lignocellulosic biomass through biochemical conversion processes involving disruption and disintegration of recalcitrant structures in order to open channels for enzymatic reaction processes in the material [9,13,14]. An effective pre-treatment technique is needed to liberate the cellulose from lignin, reduce cellulose crystallinity and increase cellulose porosity [1,15,16]. Various pre-treatment methods have been developed, but the choice of pre-treatment technology for a particular raw material is influenced by many factors such as the enzymatic hydrolysis step and the enzymes used [2]. Such pre-treatment methods include alkali and microwave-assisted pre-treatment, dilute acid, steam explosion, ammonia fibre explosion (AFEX), lime treatment and organic solvent treatments. Also, combinations of these methods have been studied and are still ongoing [2].

Microwave pre-treatment method is a physico-chemical process involving thermal and non-thermal effects. Microwaving has gained application in research studies because of its easy operation, high heat efficiency, reduction of process energy requirements, selective heating, etc [2]. The early discoveries of microwave pre-treatment on lignocellulosic biomass were reported by Ooshima et al. [17] and Azuma et al. [18] and since then, the technology has shown efficient applications in various ways [19,20,21] Microwave-assisted alkali breaks down the lignocellulosic biomass components by disruption of biomass structure, reduction in crystallinity of cellulose, improvement in the formation of fermentable sugars and reduction of the degradation of carbohydrates [22]. The pre-treatment process is carried out by immersing the biomass in alkaline solution and exposing the slurry to microwave radiation for varying residence time [21]. Research studies reported that alkaline reagents (sodium hydroxide) are the most effective and suitable for microwave-assisted pre-treatment [2,15]. Kashaninejad and Tabil [23] investigated the effect of microwave pre-treatment on the densification of wheat straw using dilute NaOH and Ca(OH)_2_ solutions. The results indicated that the density and tensile strength of microwave alkali pre-treated pellets were significantly higher than the untreated samples. Xu [24] reported on microwave/water alone pre-treatment on milled barley, spring wheat, winter wheat and oat straw for biogas. The results indicated that there was no improved yield on the anaerobic digestion of the biomass materials used and concluded that microwave pre-treatment may not be appropriate for milled straw varieties in biogas plants. The first study on the use of microwave heating for pre-treatment was carried out on rice straw and bagasse reported by Ooshima et al. [17]. The results showed increased enzymatic accessibilities by 1.6 and 3.2 times for rice straw and bagasse, compared to untreated samples. In addition, Rodrigues et al. [25] evaluated the potential of microwave-assisted alkali pre-treatment to improve the rupture of the recalcitrant structures of cashew apple bagasse and the results indicated that microwave residence time and power had no significant effect on the glucose yield. Combining microwave pre-treatment with ammonia successfully resulted in 48% delignification of sorghum bagasse at very low ammonia concentrations, reduced temperature and very short pre-treatment time compared with other technologies [24]. Microwave-assisted lime and microwave-alone pre-treatments were compared in wheat straw by Saha et al. [26]. Total sugar per gram straw released after enzymatic hydrolysis achieved from microwave-assisted lime pre-treatment at lower concentration and temperature with short pre-treatment time was higher than microwave-alone pre-treatment. In addition, reports from a previous study indicated that microwave heating (though at different operating parameters such as power level, residence time and temperature) could change the ultra-structure of cellulose, degrade lignin and hemicellulose in lignocellulosic biomass, and increase the enzymatic susceptibility of cellulosic biomass [1,27,28]. Also, microwave pre-treatment in the presence of water could improve the enzymatic hydrolysis of lignocellulosic biomass [27] and with alkali or acid, the results vary significantly with different feedstocks used [28]. The combination of microwave and chemical pre-treatment on different biomass as reported by several research studies indicated good sugar recovery; the chemicals used in this process include dilute ammonia, iron chloride and the common ones, alkaline and acid [24]. The chemicals assist the microwave pre-treatment method to remove lignin (alkali solution) and hemicellulose (acid solution) for cellulose accessibility [29]. The sugar yield of alkaline pre-treatment is dependent on the feedstock used. However, biomass used for pre-treatment process tends to react with some of the alkali and it leads to solubilization, swelling, increase in internal surface of cellulose, decrease in the degree of polymerization and crystallinity, and disruption of the lignin structure [14,30]. Microwave-assisted NaOH pre-treatment is commonly used as the pre-treatment chemical for lignocelluloses. This is due to its ability to delignify biomass although in large-scale processes, it may not be cost-effective [31]. Microwave-assisted KOH is not commonly used; it was used with switch grass at a very low concentration and reportedly was effective and generated high sugars during hydrolysis [32].

Biomass feedstock is bulky, loose and difficult to utilize as a fuel. The biomass has high moisture content, irregular shape and size, and low bulk density. All these factors make it difficult to handle, transport, store and utilize the biomass feedstock in its original form [33]. Some agricultural straws can be turned into forage by ensiling or making them into pellets for energy applications. Pelletizing of biomass is a primary means to achieve densification [34]. Densification increases the density of the final pellet product to 600–1200 kg/m^−3^ [23,35] for efficient transport and storage, and low moisture content (8% wet basis (w.b.)) for safe storage [35]. Densification of biomass, such as pelletizing or briquetting, increases bulk density, improves handling and storage characteristics, enhances volumetric calorific value, reduces transportation cost, improves combustion process control with coal, gasification and pyrolysis, increases the uniformity of physical properties (shape and size), provides clean and stable pellets for environmentally friendly fuel production [23,36] and can be utilized in other biomass-based conversions such as biochemical processes [37]. Cellulose, lignin, hemicellulose, extractives and non-extractives are components of lignocellulose biomass. 

However, it was observed from the research studies that there is a knowledge gap in the application of microwave-assisted alkali pre-treatment, densification and enzymatic saccharification on canola straw and oat hull. Therefore, the objective of this research were to investigate the effect of microwave-assisted alkali pre-treatment on the densification characteristics of canola straw and oat hull and the enzymatic digestibility of glucose in the microwave-assisted alkali pre-treated and microwave-alone (distilled water) samples in assessing the effectiveness of the pre-treatment process.

## 2. Materials and Methods

### 2.1. Sample Preparation

Two agricultural residues (canola straw and oat hull) were used in this study. The canola straw was collected from Black soil zone, Saskatchewan (52.78° N, 108.30° W) and oat hull was sourced from (Richardson Milling Ltd.) Martensville, SK, Canada (52.29° N, 106° W).

### 2.2. Experimental Setup

**Figure 1:**
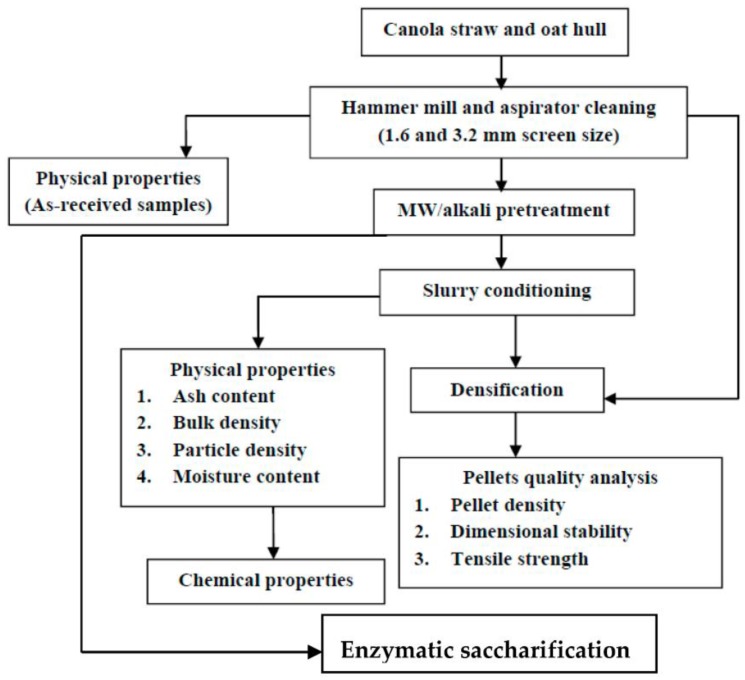
Flow diagram of the experimental procedure.

An overview of the experiment setup is shown schematically in Figure 1. After the grounding, the samples’ physical properties as received were determined and the ground samples subjected to microwave-assisted alkali pre-treatments. The sample slurries were dried and conditioned to the 12% moisture content required for densification. The physical and chemical properties of pre-treated samples were determined and the data evaluated from canola straw and oat hull pellets were analysed.

### 2.3. Cleaning, Grinding and Moisture Analysis

The canola straw was ground using a hammer mill (Glen Mills Inc. Clifton, NJ, USA) powered by a 1.5 kW electric motor with a screen opening size of 1.6 and 3.2 mm. The oat hull was cleaned using an aspirator cleaning machine (Carter-Day Company N.E, Minneapolis, MN, USA) to remove some oat kernel remaining after initial cleaning by the producers. The cleaned oat hull was ground using the same hammer mill and screen opening sizes. A dust collector including a cyclone system was used to collect the ground samples and reduced the dust during operation. The moisture contents of samples as received and ground were determined using ASABE standard S358.2 [38] in three replicates and moistures are expressed in percent wet basis (% w.b.).

### 2.4. Bulk and Particle Density Analysis

The bulk densities of pre-treated and untreated ground samples were determined and calculated using the mass and volume of a standard cylindrical steel container with 0.5 L volume (SWA951, Superior Scale Co. Ltd., Winnipeg, MB, Canada). The sample passed through a funnel and filled the 0.5 L volume container. A thin steel rod was used to roll across the top of the container in a steady pattern motion and weighed. The particle densities of the treated and untreated ground samples were determined. Ground canola straw and oat hull of known mass were placed in the gas multi-pycnometer (QuantaChrome, Boynton Beach, FL, USA) and the volume of the sample determined. Thereafter, the particle densities were calculated by mass per unit volume of the samples following the method reported by Adapa et al. [39]. The procedure was done in five replicates for both bulk and particle density measurements.

### 2.5. Particle Size Analysis 

The particle size analysis of the ground samples was determined before microwave-assisted alkali pre-treatment and densification. The geometric mean particle diameter of ground sample canola straw and oat hull was determined using ASAE Standard S319 [40]. The geometric mean diameter (*d_gw_*) of the sample and geometric standard deviation of particle diameter (*S_gw_*) were calculated using the standard mentioned [35,37,39]

### 2.6. Microwave Pre-Treatment

**Figure 2:**
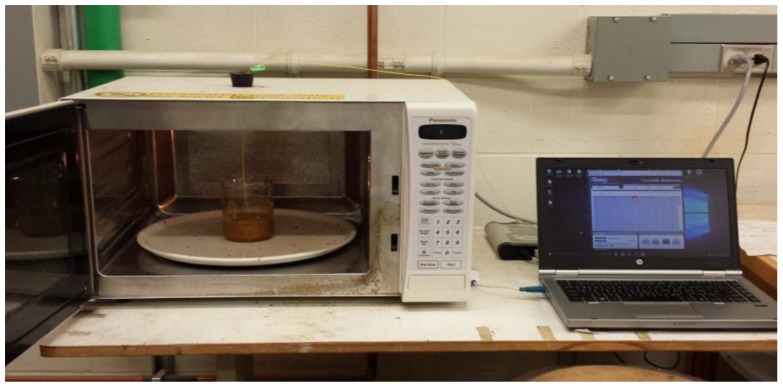
Glass beaker reactor containing the biomass–alkaline solution mixture with the temperature probe fibre in the microwave oven.

Microwave (MW) treatments were carried out using a domestic microwave oven (Model NNC980W, Panasonic Canada Ltd., Mississauga, ON, Canada) with an operating frequency of 2450 MHZ and variable power from 220 to 1100 W. The microwave heating temperature data recording and acquisition in the experiment was done using Qualitrol Corporation software and the Nomad Fiber Optic Thermometer (Model NMD228A, Quebec City, QC, Canada). The data logging was one data point for every 5 s. Twenty grams of ground biomass sample (canola straw and oat hull) were immersed in 180 g of various alkaline solutions of 0%, 0.75% and 1.5% (*w*/*v*) NaOH and 0%, 0.75% and 1.5% (*w*/*v*) KOH. The mixture was placed in a 600-mL beaker and the biomass mixture was allowed to absorb the alkaline solution for a period of 30 to 45 min. The mixture was placed at the centre of a rotating ceramic plate inside the microwave oven for treatment at a fixed power of 713 W [23]. The temperature probe was inserted through a hole closed with a cork on top of the microwave oven and inserted halfway into the beaker containing the sample. The mixture was exposed to three levels of residence time 6, 12 and 18 min, and the temperature reading was recorded accordingly. The process was done in five replicates for each sample. Figure 2 shows the experimental setup. At every interval of 3 min, the microwave was stopped, and the beaker taken out and stirred for a few seconds. This is to ensure uniform heating within the reactor. After the treatments, the moisture content of each sample was determined. The samples were dried and maintained at an appropriate moisture level of 12% (w.b.) using a forced-air convection dryer set at 42 °C [41] and stored in a Ziploc bag.

### 2.7. Chemical Analysis

The chemical composition analysis of microwave-alkali pre-treated and microwave-alone pre-treated canola straw and oat hull was performed using the National Renewable Energy Laboratory (NREL) standard [42]. The samples selected for the analysis were based on the parameters that describe pellets quality. The selection was based on tensile strength, dimensional stability and pellet density (Table not shown in this paper).

The analysis protocol according to NREL was done in two stages of acid hydrolysis, with 72% H_2_SO_4_ and 4% H_2_SO_4_, in order to fractionate the biomass into forms that are quantifiable [41]. Prior to this analysis, the biomass samples at 11%–12% (w.b.) were dried at 105 °C in an air oven (Thermo Science model No. PR305225M; Marietta, OR, USA) for 24 h. The extractive removal was done by adding the sample to a filter paper pouch, refluxed with acetone using a Soxhlet apparatus heat for 24 h. The acetone washed sample was left at room temperature for about 3–4 h in order to allow acetone to evaporate and then oven dried at 105 °C for 24 h. Acid-insoluble lignin content was evaluated based on NREL protocol as presented in Equation (1):

(1) $$\mathrm{Insoluble}\;\mathrm{lignin}\;\mathrm{content}=\frac{\left(\mathrm{dried}\;\mathrm{retentate}\right)}{\left(\mathrm{dried}\;\mathrm{sample}\right)}\times 100\%$$

The acid soluble lignin was measured using UV–Vis spectroscopy (BIOMATE 3S, Thermo Fisher Scientific, Madison, WI, USA) at an absorbance of 240 nm. Thirty millilitres of the hydrolysate were neutralized by adding 1 g of CaCO_3_ and mixed. Monosaccharide quantified using the Water Acquity UPLC–MS system (Acquity 2004–2010, Water Corp., Milford, MA, USA). Sample preparation for monosaccharide quantification was: 100 μL of stored neutralized hydrolysate with 800 μL of 75% acetonitrile/25% methanol and 100 μL of fucose solution (~1 mg/mL) and filtered through 0.2 μm filter into a 2 μL UPLC vial. The LC conditions for the monosaccharide quantification were: Acquity UPLC BEH Amide column (1.7 μm pore size, 2.1 × 50 mm); 0.25 mL min^-1^ flowrate; mobile phase A: 95% acetonitrile/5% isopropanol; mobile B: 80% acetonitrile/0.1% NH_4_OH; gradient of 100% A to 100% B over 10 min, then gradient of 100% B to 100% A over 4 min (14 min total run time per sample). The UPLC-MS conditions for the same monosaccharide quantification were: 2.8 KV; 25 V (cone); 50 L h^-1^ (cone); gas flow 600 L h^-1^; desolvation temperature 350 ^o^C; source temperature 120 ^o^C; and dwell time 0.08 s.

At the concentrated acid stage, the polymeric carbohydrates (cellulose and hemicelluloses) were hydrolyzed into monomeric forms (xylose, arabinose, mannose, glucose and galactose), soluble in the hydrolysis liquid and were measured by UPLC. The standards of the monomeric sugars were prepared and evaluated using the UPLC. The spectra of mannose, glucose, and galactose displayed at a molecular weight 179.2 g/mol while xylose and arabinose displayed at a 149.1 g/mol. The correlated monosaccharide peak extracted from the integrated peak area was used to pre-determine regression equations from dilution series of the monosaccharide standards using Microsoft Excel. 

The monomeric sugars regression analysis was determined using regression approach, the sugar content evaluated as:

(2) $$\mathrm{Sugar}\;\mathrm{content}=\frac{\mathrm{sugar}\;\mathrm{concentration}\times 87\;\mathrm{mL}\times 10\times H}{\mathrm{dried}\;\mathrm{sample}\;\mathrm{mass}}\times 100\%$$

where, *H* could be 0.88 or 0.90 depending on the number of carbons present in the sugars, which accounts for the water molecule added during the hydrolysis. The 5-carbon (pentoses: xylose and arabinose) and 6-carbon sugar (hexoses: mannose, galactose and glucose) values were multiplied by anhydro correction factors of 0.88 and 0.90, respectively, and replicated three times for each sample.

### 2.8. Ash Content

Ash content is a measure of the mineral content and extractable in biomass [41]. The ash contents of canola straw and oat hull were determined based on a National Renewable Energy Laboratory standard [43]. First, 2.0 ± 0.2 g of the oven-dried microwave alkali treated and untreated samples were weighed into the tared dried crucible. The weighed crucible and sample were placed in a muffle furnace (Model F-A1T30, Thermolyne Sybron Corp., Dubuque, IA, USA) and allowed to stay overnight at 575–600 °C. The sample was removed and placed in an oven of temperature 105 °C for 20–30 min before being placed in a desiccator to cool. The crucible and the ash were weighed. The ash content was calculated as the percentage of residue remaining after drying and each sample was replicated three times.

### 2.9. Densification

**Figure 3:**
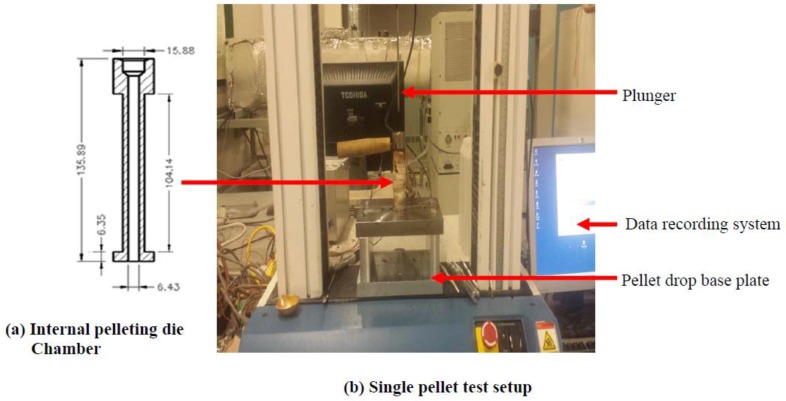
Single pellet test setup; (**a**) Internal sectional view of the single pelleting die unit (dimensions in mm) fixed in the (**b**) Instron universal tester.

The microwave-assisted alkali pre-treated and untreated samples were compressed and pelleted in a single pelleting unit consisting of a plunger-cylindrical die connected to a computer that interprets and records the force-displacement data (Figure 3). The plunger was connected to the Instron universal machine (NVLAP Lab Code 200301-0, Norwood, MA, USA), in which the upper moving crosshead provided the load necessary to compress the biomass samples. About 0.5–0.8 g of selected pre-treated and untreated biomass samples was loaded into the die cylinder. The temperature adjusted at about 95 °C and a pre-set load compressed the samples. A 5000 N load cell fitted Instron universal machine was used and a pre-set load of 4000 N compressed the samples. The plunger compressed the biomass sample using a crosshead speed of 50 mm/min. Once the pre-set load was achieved, the plunger was stopped and held in position for 60 s to avoid spring back effect of biomass [23,35]. Ten pellets each were produced from pre-treated and untreated biomass samples, and the force-deformation data at compression and the force-time data at stress relaxation were recorded in the computer. The physical characteristics of the densified pellets such as pellet density, dimensional stability, and tensile strength were determined to evaluate the effect of the treatment combination of the various factors.

### 2.10. Pellet Density and Dimensional Stability

The height, diameter, and mass of pelleted samples from microwave-assisted alkali pre-treated and untreated straws were measured immediately after pelleting using digital callipers to calculate the volume and pellet density of the samples. The pellets were stored in Ziploc plastic bags at room temperature at both stages for further analysis. After two weeks, the diameter, height and mass of the pelleted samples were measured to calculate the dimensional stability of the pellets [23]:

(3) $$\mathrm{dimensional}\;\mathrm{stability}=\frac{Vol_{14}-Vol_{0}}{Vol_{0}}\times 100\%$$

$Vol_{0}$ = Volume of pellets immediately after pelleting (mm^3^); $Vol_{14}$= Volume of pellets 14 days after pelleting (mm^3^).

### 2.11. Tensile Strength Test

The diametral compression test, as reported by Tabil and Sokhansanj [44] and Kashaninejad et al. [45], was used to determine the tensile strength of microwave-assisted alkali pre-treated and untreated canola straw and oat hull pellets. The pellets were cut diametrally into specimens of thickness about 2.5 mm using laser cutting machine. The single-cut pellet was placed at the middle of padded plate fastened on the Instron machine (Figure 3) and compressed by an upper plunger until failure occurred. The Instron was fitted with a 5000 N load cell and the samples were compressed at a crosshead speed of 1 mm/min. The specimen fractured, cracking in half, and failure occurred along the axis [46,47]. Thirteen replicates were made for each sample. The fracture force was recorded and the tensile strength calculated as:

(4) $$\delta_{x}=\frac{2F}{\pi dl}$$

where *δ_x_* is the tensile strength (horizontal) stress (Pa); *F* is the load at fracture (N); *d* is the specimen diameter (m) and *l* is the specimen thickness (m).

### 2.12. Enzymatic Saccharification

The enzymes used were cellulase (C2730-50 mL, cellulase from *Trichoderma reesei* ATTC 26921, Sigma-Aldrich Co., St. Louis, MO, USA) and β-glucosidase (C6105-50 mL, Novozyme 188, Sigma-Aldrich Co.). The addition of β-glucosidase was necessary to mitigate cellobiose inhibition of cellulase; cellobiose is a disaccharide consisting of two glucose molecules linked by a β-1, 4-glycoside bond [1,48]. To determining the cellulose activity in a suitably diluted sample, the filter paper assay was done to ascertain the filter paper unit (FPU) of the cellulase enzyme (Equation (5)) to be used in evaluating the average of one μmole of glucose equivalents released per min in the assay reaction [49] and the enzymatic saccharification analysis was performed using the dinitrosalicylic acid (DNS) method for estimating reducing sugar [50].

(5) $$\mathrm{FPU}/\mathrm{mL}=\left(\frac{A_{540\;\dot{Sample}}}{A_{540}/\mathrm{mg}\;standard}\right)\left(5.55\;\mu \mathrm{mole}/\mathrm{mg}\right)\times (\frac{1}{60\;\min})(\frac{1}{X\;\mathrm{ml}})$$

where FPU/mL is the determined cellulose activity; *A*_540_ sample is the absorbance obtained from the DNS assay for each cellulase assay; *A*_540_/mg standard is the absorbance for 1 mg of glucose measured from the glucose standard curve; 5.55 μmole/mg is the number of μmole of glucose in 1 mg; 60 min assay incubation time, and *X* mL (0.02 mL) volume of suitably diluted cellulase that was assayed.

The enzyme mixture for the saccharification assay was prepared in a 10-mL clear scintillation vial tube such that 0.25 mL enzyme contains 85.54 FPU/mL cellulase (0.93 mL), 300 cellobiase units CBU/mL Novozyme 188 (0.53 mL) and 0.54 mL sodium acetate buffer (50 mM, pH 4.8) for the digestion.

One gram dry biomass sample was weighed and transferred into 50-mL flasks containing 19.75 mL sodium acetate (NaAc) buffer (50 mM, pH 4.8). The pH reading for enzymatic cellulose saccharification of lignocellulosic substrates was determined and in range with the substrate suspension pH 5.2–6.2 [51]. To each flask, 100 μL of a 2% sodium azide solution were added and this was used to prevent microbial growth during digestion. The reactions were setup according to NREL protocol [42,49]. A 20 μL aliquot was collected and prepared for micro-plate DNS glucose analysis. Three replicates of each sample were performed.

### 2.13. Micro-Plate DNS Glucose Analysis

The glucose (total reducing sugar) was analysed using the micro-plate modified DNS assay as described [49,50]. In order to determine the standard curve of the amount of glucose in each well, 60-μL format assay was used. The reason is because it is highly reproducible, accurate and van easily assay a large number of samples compared to standard and 96-μL filter paper assay protocols [52]. Using the 60 μL format assay, a 20 μL aliquot of sample was added into PCR micro-plate (Thermowell Fisher, Ottawa, ON, Canada) wells containing 40 μL of 50 nM NaAc buffer (pH 4.8) and 120 μL DNS solution was added to each well. The plate was covered with the thermowell sealer and incubated at 95 °C for 5 min. After incubation, a 36-μL aliquot of each digested sample was transferred to a 96-well flat-bottomed micro-plate (Corning Inc., Corning, NY, USA) containing 160 μL of water and *A*_540_ nm measured (Spectra Max-Plus, Sunnyvale, CA, USA). The mean *A*_540_ was used to calculate the expected *A*_540_ for 1 mg glucose digested. 

The glucose digestion percentage was calculated using Equation (6):

(6) $$\mathrm{Glucose}\;\mathrm{digestion}\;\%=\frac{\mathrm{cellulose}\;\mathrm{digested}\;\left(\mathrm{mg}\right)}{\mathrm{cellulose}\;\mathrm{added}\;\left(\mathrm{mg}\right)}\;\times \;100$$

### 2.14. Scanning Electron Microscopy

Physical and structural changes on the cell walls of ground untreated, microwave-alone and microwave-assisted alkali pre-treated canola straw and oat hull were observed by JEOL, JSM-6010LV scanning electron microscope (SEM) (JEOL USA, Inc., Peabody, MA, USA) taken at magnifications of 250 and 500×. Prior to the image examination, the samples were coated with a thin conducting layer of ~10–100 nm gold sputter. The coating was achieved through sputtering by plasma under a vacuum (Model S150B, Sputter Coater, Edwards, NY, USA). This is to improve the sample electronic conductivity during imaging [53]. The fine-coated specimens were fixed on the stub with adhesive and observed using a voltage of 5 kV.

### 2.15. Statistical Analysis

Response Surface Methodology (RSM) is a statistical technique for designing experiments, building models, evaluating the effects of factors that extract the maximal information with the minimal number of runs [54,55]. In order to statistically study the effect of microwave treatment and alkali solution, User-Defined Design (UDD) was applied via analysis of variance (ANOVA) to investigate the effect of microwave heating time and alkali concentration on the compaction of canola straw and oat hull. The range and levels of variables determined are shown in Table 1 and a polynomial quadratic equation was fitted to evaluate the effect of each independent variable against the responses:

(7) $$y_{n}=\beta_{0}+\sum_{i=1}^{2}\beta_{i}x_{i}+\sum_{i=1}^{2}\beta_{ii}x_{i}^{2}+\sum_{i=1}^{2}\sum_{j=i+1}^{2}\beta_{ij}x_{i}x_{j}\;(n=1,\;2,\;3,\;4,\;5,\;6)$$

where *x*_1_ the alkali concentration (%) and *x*_2_ the microwave heating time (min) are the independent variables which influence the response variables *y* (pellets density (kg/m^3^), dimensional stability (%), tensile strength (MPa), ash content (%), bulk density (kg/m^3^), particle density (kg/m^3^); β_0_ the offset term, β_i_ is the *i*th linear term, β_ii_ is the quadratic term and β_ij_ is the *ij*th interaction terms in the equation. The response surfaces of the variables in the experimental design domain were analysed by Design Expert software (Version 8.0.7.1, Stat-Ease Inc., Minneapolis, MN, USA, 2010).

## 3. Results and Discussion

### 3.1. Physical Properties

Table 2 shows the physical properties of ground canola straw and oat hulls. The geometric mean particle diameter of canola straw was slightly smaller than that of oat hull samples. The ash content was higher in canola straw samples compared to oat hull samples. This may be due to the variation in moisture content and mechanical properties of the different biomass. The canola straw ground in the hammer mill using a 1.6-mm screen size was the finest among the ground biomass. However, the oat hull sample ground in the hammer mill with a 1.6-mm screen had the highest bulk and particle densities of 331.32 and 1440.51 kg/m^3^, respectively. Samples ground in the hammer mill using a large screen size, e.g., 3.2 mm, resulted in lower bulk and particle densities.

Table 3 and Table 4 are the physical properties of microwave-assisted alkali pre-treated canola straw (CS) and oat hull (OH), respectively. It was observed that samples pre-treated with microwave alone showed lower bulk and particle densities (108.10 kg/m^3^ and 982.42 kg/m^3^, respectively) than the untreated samples (Table 2). Increasing the time and alkali concentration increased the ash content and bulk density of microwave-alkali pre-treated canola straw and oat hull. The analysis of variance of the data shows that microwave heating time and alkali concentration significantly affected the bulk density of microwave-alkali pre-treated canola straw and ash content of microwave-alkali pre-treated oat hull in treatments NaOH and KOH/1.6 and 3.2 mm, and microwave heating time had a significant effect on the bulk density of microwave-alkali pre-treated oat hull. Similarly, increasing the alkali concentration increased the particle density for microwave-alkali pre-treated canola straw and oat hull except at 3.2 mm 0.75% NaOH. The microwave heating time did not have a significant effect on particle density for microwave-alkali pre-treated oat hull and canola straw. The alkali concentration and microwave heating significant effects in the pre-treated samples were the result of microwave pre-treatment, which causes the swelling of the material and increases the internal surface area of lignocellulosic structures [23]. (The ANOVA table is not included in the paper.) Canola straw and oat hull pre-treated by microwave-assisted alkali showed higher bulk and particle densities than untreated samples. Kashaninejad and Tabil [23] reported that this is a result of increased depolymerized components and ash content of pre-treated samples. In addition, samples pre-treated with microwave/NaOH had higher bulk and particle densities than samples pre-treated with microwave/KOH.

### 3.2. Chemical Composition of Microwave-Assisted Alkali Pre-Treated Canola Straw and Oat Hull

Table 5 shows the lignocellulosic composition of microwave-assisted alkali pre-treated and microwave alone pre-treated canola straw and oat hull samples. Reports from previous studies stated that alkali treatments dissolve lignin and hemicellulose, and microwave heating enhances the breakdown of these components in alkali solutions [23,56]. The cellulose content increased with increasing alkali concentration and microwave heating time, whereas the lignin content decreased with increase in microwave heating time and alkali concentration. This implies that there is a breakdown of the biomass matrix in the lignin and creates the accessibility and digestibility of cellulose and hemicellulose [30,56]. The lignin content of pre-treated canola straw and oat hull samples was lower than microwave-alone pre-treated samples, except in treatment CS 1.6 mm 0.75% KOH/12 min. The lignin decrease shows an indication of solubilisation in the alkaline aqueous solution and an increase in cellulose was as a result of solubilisation from other components in the alkali solution. Also, increase in cellulose content by microwave heating was facilitated by the dissolution of components in alkaline solutions [15,57]. The microwave-assisted alkali pre-treatment removed more hemicellulose and lignin in canola straw than oat hull samples. In addition, microwave-alkali in both feedstocks resulted in higher solubilisation of cellulose and, decrease in hemicellulose and lignin. The stronger alkaline pre-treatment in combination with long microwave heating time caused more solubilisation of cellulose, hemicellulose and lignin. Zhu et al. [57,58] reported a similar result with wheat straw and rice straw. For both feedstocks in this study, canola straw samples showed higher solubilisation with the alkali solution than the oat hull in microwave-assisted alkali pre-treatment. This shows that the alkaline used in the pre-treatments caused swelling and lignin structure disruption in the biomass that resulted in the solubility of lignin in the samples [30,59]. Kashaninejad and Tabil [23] reported that the main aim of using the alkali solution during the microwave pre-treatment method is to disintegrate the ester bonds between lignin and carbohydrate in the biomass; this statement is supported with the data presented herein.

### 3.3. Pellet Density

Table 6 and Table 7 show the effect of microwave-alkali pre-treatments on pellet density, dimensional stability and tensile strength for canola straw and oat hull pellets. The surface of microwave-alkali pre-treated samples appeared smoother and darker than alkali-treated and untreated samples, and Kashaninejad and Tabil [23] reported similar results with pellets produced from wheat and barley straw grinds. The microwave-assisted alkali pre-treated samples showed the highest pellet density (canola straw 1392.21 kg/m^3^ and oat hulls 1292.59 kg/m^3^) compared to microwave-alone and untreated samples. Increasing the alkali concentration increased the pellet density of the samples. Increasing the microwave heating time decreased the pellet density of canola straw samples with treatments of 1.6 mm/0% 0.75% and 1.5% KOH and 3.2 mm/0% and 0.75%; for oat hull, the microwave heating time increased in treatments of 1.6 mm/1.5% NaOH, 0.75 and 1.5% KOH and decreased in treatment 3.2 mm/0.75% KOH. Iroba et al. [60] reported that samples release binding agent (lignin) which increases adhesion within the particles, activates the intermolecular bonds within the contact area of the samples, and improves the mechanical interlocking of the particles during pelleting.

Analysis of variance of the data (Table 8 and Table 9) shows that the alkali concentration significantly (*p* < 0.05) affected canola straw and oat hull pellet density. Microwave heating time had a significant effect for samples with treatments of KOH/1.6 mm for canola straw and oat hull pellets. Microwave/NaOH pre-treatment was more effective at the initial heating time for 0.75% alkali concentration in increasing the initial density of the pellets, while microwave/KOH pre-treatment was more effective at1.5% alkali concentration in increasing the initial pellet density.

### 3.4. Dimensional Stability 

The dimensional stability values for canola straw and oat hull pellets were evaluated from the pellet’s dimensional measurements after 14 days and are presented in Table 6 and Table 7. Samples pre-treated with microwave-assisted alkali have the highest dimensional stability (close to 0), as compared to samples pre-treated with microwave heating only and untreated samples. In canola straw, microwave-assisted alkali pre-treated canola straw and oat hull pellets had the highest dimensional stability in 3.2 mm 0.75% NaOH and KOH at 6 min. This is because samples released the binding agent (lignin), which increased the adhesion within the particles, activated the intermolecular bonds within the contact area of the samples and enhanced the mechanical interlocking of the particles [60]. The data indicated that the dimensional stability of canola straw pellets decreased with an increasing alkali concentration in the following treatments: 6 and 18 min/NaOH 1.6 mm screen size; 12 and 18 min/NaOH and KOH 3.2 mm screen size. Oat hull pellet dimensional stability decreased with increasing alkali concentration in the following treatments: 18 min/NaOH; 6 and 18 min/KOH 1.6 mm screen size; 12 and 18 min/NaOH and 18 min/KOH 3.2 mm screen size. Lower microwave heating time resulted in higher stability of the canola straw pellets for treatment combination of: 1.6 mm/0; 3.2 mm/0%and 1.5% KOH and in oat hulls pellets 1.6 mm/0.75% and 1.5% KOH, and 3.2 mm/0%, 0.75 and 1.5% NaOH and KOH. Iroba et al. [60] and Tabil [61] reported that when the biomass is heated, the lignin becomes soft, melts and exhibits thermosetting binder resin properties to produce pellets with higher density and dimensional stability.

Analysis of variance (Table 8 and Table 9) shows that alkali concentration and microwave heating time significantly (*p* < 0.05) affected the dimensional stability of the canola straw and oat hull pellets in 3.2 mm screen size for both NaOH and KOH and 1.6 mm canola straw. In the other treatments, only microwave heating had a significant effect on the pellet stability of oat hull 3.2 mm KOH. From analysis of variance, both alkali concentration and microwave heating time affected the dimensional stability of microwave-assisted alkali pre-treated canola straw and oat hull pellets. Pellets produced from microwave-alkali pre-treated samples will present easy handling and storage and result in efficient transportation in terms of withstanding shear, impact, rotation and tumbling with minimal generation of fine particulate matter [35,39,60].

### 3.5. Tensile Strength of Pellets

Table 6 and Table 7 show the tensile strength (evaluated using Equation (5) and the fracture load values of the pellets produced from microwave-assisted alkali pre-treated, microwave-alone and untreated canola straw and oat hull which were evaluated using Equation (5). The observed data indicate that alkali concentration and microwave heating time are important factors for the physical characteristics of the pellets. Microwave-assisted-alkali pre-treated pellet samples showed highest tensile strength (canola straw = 5.22 MPa at 1.6 mm 1.5% NaOH 6 min and oat hull = 3.36 MPa at 1.6 mm 1.5% NaOH 18 min). Increasing the alkali concentration increased the tensile strength of canola straw and oat hull pellets. This means that microwave-assisted alkali pre-treatment has the ability to disintegrate the structure of lignocellulosic biomass involved in particle binding [23] and results in the breakdown of lignin components. Thus, the lignin after pre-treatment assisted in the particle binding mechanisms during pelletizing resulting in pellets with higher tensile strength and fracture load [60]. Longer microwave heating time resulted in lower tensile strength of canola straw pellets but higher tensile strength of oat hull pellets in treatments combinations of: 1.6 mm/1.5% NaOH and KOH; 3.2 mm/0%, 1.5% NaOH and, 0.75%; 1.5% KOH. Samples pre-treated with microwave alone (MW/distilled water) have lower tensile strength and fracture load than others. This is because water and heat alone are not sufficient to disintegrate the lignocellulosic matrix of biomass [60].

Analysis of variance performed on the data (Table 8 and Table 9) shows that alkali concentration and microwave heating time had significant effects (*p* < 0.05) on the tensile strength of canola straw pellet in treatments of 1.6 mm NaOH/KOH and 3.2 mm KOH. The same was true for oat hull pellets in treatments of 3.2 mm NaOH. Consequently, only the alkali concentration had a significant effect on the tensile strength of canola straw and oat hull pellets.

**Figure 4:**
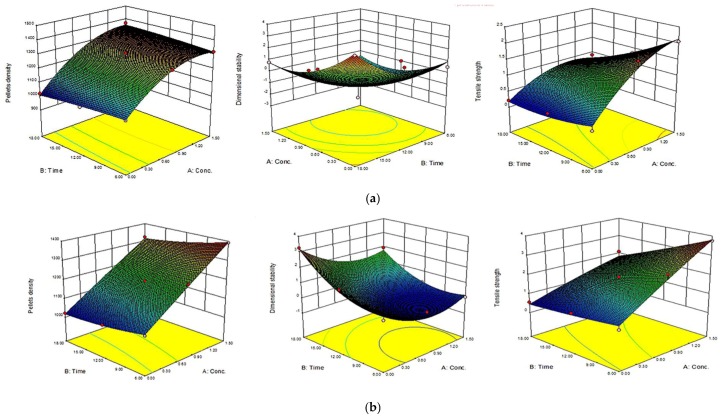
Response surface for the effects of alkali conc. (%) and MW heating time (min) on MW/alkali pre-treated 1.6 mm canola straw (CS) pellets. (**a**) using NaOH and (**b**) KOH.

**Figure 5:**
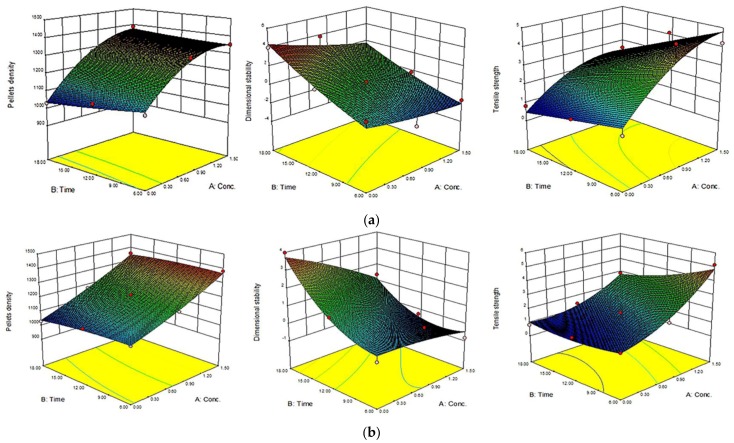
Response surface for the effects of alkali conc. (%) and MW heating time (min) on MW/alkali pre-treated 3.2 mm canola straw (CS) pellets. a) using NaOH (**a**) and b) KOH.

**Figure 6:**
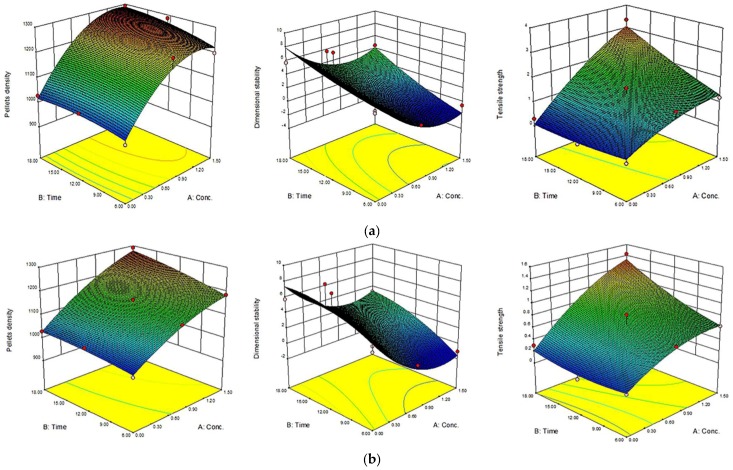
Response surface for the effects of alkali conc. (%) and MW heating time (min) on MW/alkali pre-treated 1.6 mm oat hull (OH) pellets. (**a**) using NaOH and (**b**) KOH.

**Figure 7:**
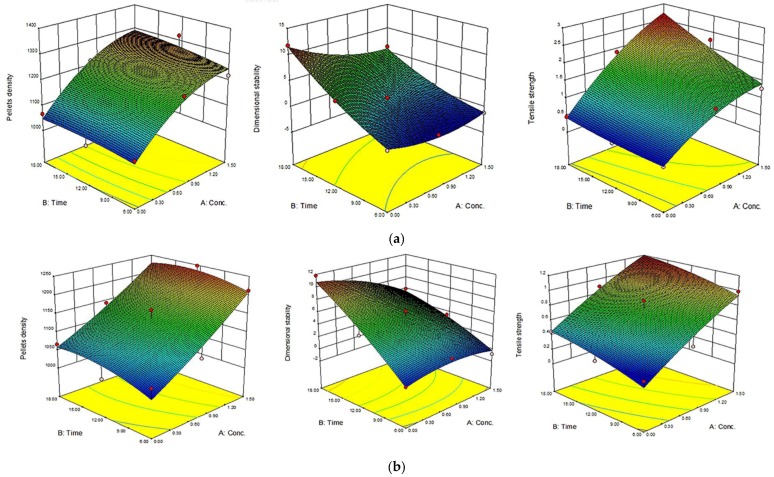
Response surface for the effects of alkali conc. (%) and MW heating time (min) on MW/alkali pre-treated 3.2 mm oat hull (OH) pellets. (**a**) using NaOH and (**b**) KOH.

The 3D response surface and the 2D contour plots of the responses from microwave-assisted pre-treated canola straw and oat hull pellets are shown in Figure 4, Figure 5, Figure 6 and Figure 7. In order to depict the interactive effects, each of these responses’ pellet density, dimensional stability and tensile strength were kept constant while the two independent variables (alkali concentration and MW heating) varied within certain ranges. The response surfaces and contour plots of the samples differ according to the alkali solution used in the study. Comparatively notable interactions among the variables were shown in microwave/NaOH pre-treated samples by their shapes and contours compared to KOH pre-treated samples. The interaction among the pellet density, dimensional stability and tensile strength significantly influenced the pellet quality of the samples regardless of the alkaline concentration and microwave heating time of the samples.

Furthermore, high alkali concentration with long microwave heating time resulted in high pellet density in canola straw and oat hull pellets. However, negligible interactions were shown with dimensional stability of the samples’ pellets by the irregular shape of the contour plots (1.6 mm CS and OH/3.2 mm OH KOH), while comparatively prominent interactions were shown with pellets’ densities and tensile strengths by the rectangular curved nature of the contour plots of the samples. In other words, the interaction effects of alkali concentration and microwave heating time significantly affected the physical qualities of canola straw and oat hull pellets.

### 3.6. Glucose Yield

Microwave-assisted alkali and microwave alone pre-treated canola straw and oat hull samples were used as the substrates for enzymatic saccharification. These substrates were subjected to enzymatic saccharification in order to convert cellulose to glucose and saccharification of cellulosic biomass prior to fermentation to ethanol; this is a very important step because the yeast (*S. cerevisiae*) used is a non-amylolytic microbe [1]. Table 10 show the glucose yields in one gram of the dry biomass samples (canola straw and oat hull). The data validates the effectiveness of the pre-treatment method by reflecting the accessibility and digestibility of cellulose (glucose) in the microwave-assisted alkali pre-treated samples compared to microwave-alone pre-treated samples.

The highest glucose (sugar) yield (110.05 mg/g) for one gram dry canola straw sample was obtained from microwave-assisted alkali pre-treatment with 1.5% NaOH for 18 min; the sample was ground using a 1.6 mm hammer mill screen size. For 1.5% and 0.75% NaOH and KOH treatments, the yield significantly increased with longer microwave heating time for canola straw ground using 1.6 mm screen size, whereas for those ground with the 3.2 mm screen size, the glucose yield significantly increased with shorter microwave heating time. 

The highest glucose yield (99.10 mg/g) for one gram dry oat hull sample was obtained from microwave-assisted alkali pre-treatment with 0.75% NaOH for 18 min; the sample was ground using a 1.6 mm hammer mill screen size. Sugar yields increased as the microwave heating time was extended from 6 to 18 min with treatment with 0.75% NaOH and decreased in treatment combinations of 1.5% NaOH/1.6 and 3.2 mm hammer mill screen sizes. Moreover, treatment of 1.5% KOH using 1.6 mm hammer milled oat hull resulted in significantly increased sugar yields with lower microwave heating time. 

In addition, microwave alone (distilled water) pre-treated samples showed lower glucose yield compared to microwave-assisted alkali pre-treated samples. The glucose yield in microwave alone pre-treatment increased as the microwave heating time was extended from 6 to 18 min in both samples. Consequently, the results revealed that in 0.75% and 1.5% NaOH and KOH treatments combinations peak glucose yields were obtained at 18 min of microwave heating time for samples ground with 1.6 mm hammer mill screen size. Also, at initial microwave heating time of 6 min, glucose yield significantly increased with increasing alkali concentration in both treatments. 

Furthermore, it was observed that the effect of alkali concentration on the glucose yield varied with microwave heating time in canola straw and oat hull samples. Pre-treatment using NaOH solution at different concentrations resulted in higher glucose yields compared to KOH in both feedstocks. This implies that an NaOH solution with microwave pre-treatment was effective to delignify biomass [31]. Also, the data obtained from this investigation revealed that high glucose yields were observed in samples ground using a 1.6 mm hammer mill screen size for both feedstocks.

### 3.7. Structural Changes in the Biomass

**Figure 8:**
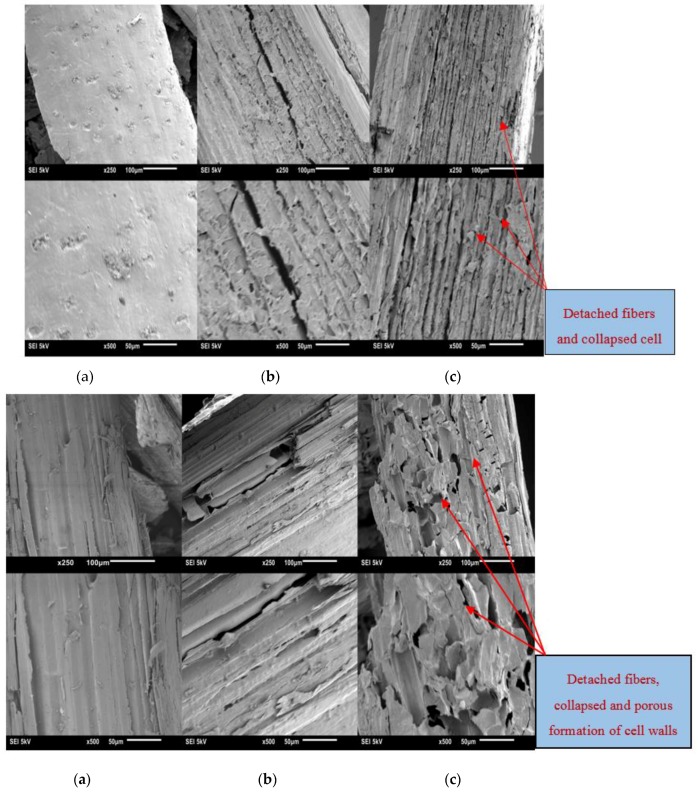
SEM images of oat hull (**a**–**c**) and canola straw (**a**–**c**) at 250 and 500× magnifications: (**a**) untreated sample; (**b**) microwave pre-treated with distilled water; (**c**) microwave-assisted alkali pre-treated.

The structural changes that were induced by microwave-assisted alkali pre-treatments were investigated by SEM. The examined images of pre-treated canola straw and oat hull were compared with microwave alone pre-treated and untreated samples at magnification of 250 and 500×. Figure 8a–c show the observed changes in untreated, microwave alone pre-treated and microwave-assisted alkali pre-treated canola straw and oat hull surfaces. The SEM in Figure 8 a showed the undamaged surface of untreated oat hull and canola straw particles, which were smooth, contiguous and intact. In Figure 8 b, the oat hull and canola straw microwaved with distilled water showed slight disorder and disruption on the surfaces compared to the untreated samples. Some opened cell walls were evident and can be recognized. Figure 8 c show that the microwave-assisted alkali pre-treated oat hull and canola straw particles have detached fibres, collapsed cell walls and with porous formation on the individual cell wall transverse plane surfaces. Similar results were reported by Anna and de Souza [62] and Diaz et al. [52].

Furthermore, the SEM images show evidence of breakdown of the lignocellulosic matrix, which is advantageous in releasing the binding agent (lignin) and activating the intermolecular bonds to improve the quality of compressed pellets [60]. Also, the images reveal that alkali solution used in the pre-treatments caused swelling and disruption of the lignin structure in the biomass, resulting in enzymatic accessibility and the digestibility of cellulose and hemicellulose [30,56].

### 3.8. Variable Optimization

The optimal condition goals for microwave-assisted alkali pre-treatment of canola straw and oat hull pellets were extracted from numerical optimization by Design Expert software. The response variables (pellet density and tensile strength) are to be maximized and dimensional stability is to be minimized. In considering the level of importance, tensile strength is the most important property due to the physical resistance of pellets to the forces in pellet handling and transportation. Dimensional stability is next, indicating less dust generation during handling, followed by pellet density because high density is another desirable property in pellet handling. Alkali concentration and microwave heating time were placed in range as shown in Table 11.

Table 12 presents the optimum operating parameters results of all the variables as extracted by the software. The results showed that a 1.5% alkali concentration was considered optimal regardless of the screen size of the hammer mill used to grind the samples, whereas a reduced microwave heating time (approximately 6 min) was considered optimal for canola straw and longer microwave heating (9–18 min) for oat hull samples in both screen sizes of hammer mill used to grind the samples. At the same time, it was observed that the optimum operating conditions selected for microwave-assisted alkali pre-treatment were best for canola straw and oat hull that were hammer milled with a 1.6 mm screen size.

## 4. Conclusions

Microwave-assisted alkali pre-treatment was found to enhance the densification and enzymatic saccharification of canola straw and oat hull. The following conclusions can be drawn from this investigation:

1. Microwave-assisted alkali pre-treatment of canola straw and oat hull resulted in better physical quality and improved the enzymatic digestibility of these substrates.
2. Canola straw and oat hull samples hammer milled with 1.6 mm screen size resulted in pellets with better physical quality compared to samples hammer milled with 3.2 mm screen size.
3. Alkali concentration of 1.5% with a microwave heating time of approximately 6 min resulted in high tensile strength of canola straw, whereas a microwave heating time of 9–18 min and alkali concentration of approximately 1.5% resulted in high tensile strength of oat hull pellets.
4. Microwave-assisted alkali pre-treatment was able to disrupt and breakdown the lignocellulosic structure of the samples and created accessible areas of cellulose for cellulase reactivity.
5. The best enzymatic saccharification result that gave a high glucose yield of 110.05 mg/g dry sample for canola straw was ground in a 1.6 mm screen hammer mill and microwave pre-treated with 1.5% NaOH for 18 min. A high glucose yield of 99.10 mg/g dry sample for oat hull resulted from those ground in a 1.6 mm screen hammer mill and microwave pre-treated with 0.75% NaOH for 18 min.
6. Structural changes of sample particles of microwave-assisted alkali pre-treated canola straw and oat hull were observed through SEM images revealing the effectiveness of microwave-assisted alkali pre-treatment.
7. Overall, microwave-assisted alkali pre-treatment of canola straw and oat hull improved biomass pellet quality and glucose (sugar) yield for bioethanol production.

## Figures and Tables

**Table 1:** Code levels for independent variables used in the UDD and actual factor levels corresponding to coded factor levels.

Independent Variable	Code	Actual Factor Level at Coded Factor Levels		
		−1	0	1
A—Alkali conc. (%)	*X*_1_	0	0.75	1.5
B—MW time (min)	*X*_2_	6	12	18

**Table 2:** Physical properties of ground canola straw and oat hull.

Sample	Screen Size (mm)	Moisture Content as Received ^a^ (% w.b.)	Moisture Content Ground Sample ^a^ (% w.b.)	d_gw_^a^ (mm)	S_gw_^a^ (mm)	Ash Content ^a^ (%)	Bulk Density ^b^ (kg/m^3^)	Particle Density ^b^ (kg/m^3^)
Canola straw	1.6	9.08 ± 0.45	7.64 ± 0.59	0.348 ± 0.02	0.280 ± 0.01	6.47 ± 0.87	168.14 ± 2.67	1305.53 ± 46.08
	3.2		8.28 ± 0.39	0.520 ± 0.04	0.498 ± 0.01	6.66 ± 1.56	141.16 ± 2.10	1220.41 ± 6.20
Oat hull	1.6	9.72 ± 0.15	6.96 ± 0.33	0.370 ± 0.00	0.217 ± 0.01	5.31 ± 0.26	331.32 ± 4.39	1440.51 ± 3.25
	3.2		7.7 ± 0.12	0.547 ± 0.00	0.284 ± 0.00	5.65 ± 1.62	285.10 ± 9.16	1391.01 ± 8.40

Geometric mean diameter = d_gw_; Geometric standard deviation = S_gw_. ^a^ Mean ± standard deviation of three replicates; ^b^ Mean ± standard deviation of five replicates.

**Table 3:** Average ash content and bulk and particle densities of microwave alkali pre-treated canola straw samples.

Sample	Screen Size (mm)	Alkali	Concentration (%)	Microwave Heating Time (min)	Microwave Pre-Treated Temperature ^b^ (°C)	Ash Content ^a^ (%)	Bulk Density ^b^ (kg/m^3^)	Particle Density ^b^ (kg/m^3^)
Canola straw	1.6		0	6	92.20 ± 0.10	5.16 ± 0.29	122.43 ± 1.43	1262.91 ± 30.54
			0	12	92.33 ± 0.15	5.33 ± 0.57	134.57 ± 1.77	1206.60 ± 7.39
			0	18	112.93 ±7.39	5.48 ± 0.48	137.72 ± 1.85	1124.45 ± 8.44
		NaOH	0.75	6	72.82 ± 4.79	15.50 ± 0.50	149.41 ± 0.22	1514.18 ± 12.19
			0.75	12	75.24 ± 0.69	14.83 ± 0.28	171.54 ± 1.53	1423.10 ± 10.48
			0.75	18	82.88 ± 8.70	14.33 ± 0.28	183.15 ± 2.29	1303.16 ± 5.36
			1.5	6	75.16 ± 1.33	20.13 ± 1.02	160.22 ± 2.48	1572.56 ± 81.84
			1.5	12	77.20 ± 1.36	22.17 ± 0.57	194.72 ± 2.22	1472.80 ± 2.98
			1.5	18	78.90 ± 1.10	22.96 ± 0.07	260.11 ± 0.90	1358.89 ± 5.27
		KOH	0.75	6	76.62 ± 2.61	12.83 ± 0.58	137.79 ± 0.89	1389.39 ± 8.86
			0.75	12	76.76 ± 1.55	12.67 ± 0.28	154.45 ± 1.55	1428.87 ± 13.54
			0.75	18	78.72 ± 1.65	12.33 ± 0.28	157.00 ± 0.73	1134.54 ± 9.08
			1.5	6	90.30 ± 1.33	19.33 ± 0.28	145.33 ± 0.75	1411.14 ± 3.02
			1.5	12	91.76 ± 0.24	19.67 ± 0.57	173.98 ± 2.46	1496.22 ± 8.69
			1.5	18	116.92 ± 3.38	19.83 ± 0.29	200.99 ± 2.06	1343.62 ± 7.44
	3.2		0	6	92.07 ± 0.23	5.17 ± 0.28	108.10 ± 1.52	1033.48 ± 11.03
			0	12	92.23 ± 0.06	5.33 ± 0.28	116.40 ± 1.09	1045.16 ± 16.35
			0	18	116.67 ± 10.47	5.50 ± 0.50	126.96 ± 3.56	982.42 ± 20.54
		NaOH	0.75	6	91.60 ± 0.10	15.17 ± 0.29	131.71 ± 3.05	1324.92 ± 5.20
			0.75	12	92.04 ± 0.36	14.67 ± 0.29	148.11 ± 1.83	1423.39 ± 18.12
			0.75	18	119.02 ± 8.19	14.33 ± 0.58	170.48 ± 2.18	1229.76 ± 20.79
			1.5	6	90.36 ± 0.90	21.17 ± 0.58	153.09 ± 2.68	1462.90 ± 2.73
			1.5	12	91.90 ± 0.21	22.33 ± 0.28	182.61 ± 3.74	1466.03 ± 4.01
			1.5	18	123.26 ± 8.18	22.50 ± 1.00	247.76 ± 3.24	1297.84 ± 19.89
		KOH	0.75	6	91.24 ± 0.09	13.17 ± 0.28	114.86 ± 3.16	1285.89 ± 11.96
			0.75	12	91.38 ± 0.11	13.00 ±0.00	133.13 ± 2.98	1335.35 ± 9.93
			0.75	18	124.32 ± 6.98	12.67 ± 0.58	137.40 ± 1.70	1043.47 ± 5.22
			1.5	6	91.04 ± 0.08	20.17 ± 0.76	123.82 ± 1.12	1429.45 ± 6.89
			1.5	12	91.42 ± 0.24	20.50 ± 0.50	164.70 ± 3.58	1511.66 ± 8.47
			1.5	18	124.12 ± 5.01	20.67 ± 0.29	190.07 ± 1.94	1281.60 ± 1.84

^a^ Mean ± standard deviation of three replicates; ^b^ Mean ± standard deviation of five replicates.

**Table 4:** Average ash content and bulk and particle densities of microwave alkali pre-treated oat hull samples.

Sample	Screen Size (mm)	Alkali	Concentration (%)	Microwave Heating Time (min)	Microwave Pre-Treated Temperature ^b^ (°C)	Ash Content ^a^ (%)	Bulk Density ^b^ (kg/m^3^)	Particle Density ^b^ (kg/m^3^)
Oat hull	1.6		0	6	92.56 ± 0.29	4.67 ± 0.28	256.60 ± 2.53	1427.75 ± 1.90
			0	12	93.03 ± 0.23	4.83 ± 0.58	264.84 ± 2.23	1430.20 ± 3.24
			0	18	93.86 ± 0.10	5.00 ± 0.50	321.27 ± 3.47	1410.10 ± 3.43
		NaOH	0.75	6	90.78 ±4.02	8.50 ± 0.50	235.95 ± 2.10	1465.14 ± 5.45
			0.75	12	92.26 ± 0.27	9.67 ± 0.29	270.10 ± 3.45	1502.91 ± 3.28
			0.75	18	106.62 ± 3.75	9.83 ± 1.26	334.46 ± 1.99	1502.89 ± 3.04
			1.5	6	88.45 ± 5.73	15.17 ± 0.29	280.39 ± 1.22	1544.32 ± 2.47
			1.5	12	92.75 ± 0.07	15.83 ± 0.28	329.28 ± 3.70	1557.82 ± 2.86
			1.5	18	104.55 ± 8.83	16.17 ± 0.28	353.11 ± 4.58	1548.69 ± 1.87
		KOH	0.75	6	92.87 ± 0.06	7.00 ± 0.00	243.14 ± 2.69	1447.42 ± 20.92
			0.75	12	92.90 ± 0.10	7.17 ± 0.76	276.28 ± 2.57	1451.40 ± 5.83
			0.75	18	111.97 ± 2.60	7.83 ± 1.04	298.96 ± 2.57	1464.83 ± 4.13
			1.5	6	92.44 ± 0.18	13.00 ± 0.50	247.12 ± 4.35	1498.86 ± 4.61
			1.5	12	92.58 ± 0.28	13.17 ± 0.28	290.26 ± 6.56	1546.26 ± 3.00
			1.5	18	114.88 ± 8.18	13.50 ± 0.87	339.04 ± 5.50	1523.19 ± 3.33
	3.2		0	6	92.53 ± 0.23	4.50 ± 0.00	207.07 ± 3.56	1373.74 ± 4.94
			0	12	92.50 ± 0.26	4.67 ± 0.28	206.46 ± 2.41	1361.42 ± 3.36
			0	18	95.00 ± 2.18	5.33 ± 0.76	240.53 ± 1.46	1394.83 ± 2.81
		NaOH	0.75	6	92.00 ± 0.20	8.83 ± 0.28	207.31 ± 1.58	1435.75 ± 6.46
			0.75	12	92.30 ± 0.36	9.00 ± 0.50	238.94 ± 5.06	1506.29 ± 2.20
			0.75	18	126.30 ± 5.98	9.50 ± 0.50	253.98 ± 4.10	1505.66 ± 3.12
			1.5	6	92.38 ± 0.22	15.00 ± 0.50	236.56 ± 3.52	1533.35 ± 3.15
			1.5	12	92.68 ± 0.59	15.67 ± 0.76	336.55 ± 2.58	1559.22 ± 1.24
			1.5	18	119.43 ± 8.42	16.00 ± 0.50	283.27 ± 4.70	1548.87 ± 1.62
		KOH	0.75	6	92.73 ± 0.24	7.17 ± 0.76	209.05 ± 3.59	1456.67 ± 2.93
			0.75	12	92.40 ± 0.18	7.50 ± 0.50	217.53 ± 2.53	1464.51 ± 2.08
			0.75	18	120.70 ± 4.24	8.00 ± 0.50	244.91 ± 3.91	1457.28 ± 3.67
			1.5	6	92.60 ± 0.36	13.33 ± 0.28	221.49 ± 4.73	1507.52 ± 4.02
			1.5	12	93.27 ± 0.06	13.67 ± 0.58	257.88 ± 2.77	1541.46 ± 1.47
			1.5	18	115.70 ± 3.40	13.83 ± 0.29	258.75 ± 5.25	1530.87 ± 2.55

^a^ Mean ± standard deviation of three replicates; ^b^ Mean ± standard deviation of five replicates.

**Table 5:** Chemical composition (% dry basis) of microwave-assisted alkali pre-treated canola straw and oat hull.

Sample	Screen Size (mm)	Alkali	Concentration (%)	Microwave Heating Time (min)	C ^a^ (%)	H ^a^ (%)	L ^a^ (%)
Canola straw	1.6		0	18	63.1 ± 32.0	5.5 ± 6.3	5.0 ± 0.8
		NaOH	1.5	18	59.1 ± 0.5	9.4 ± 8.3	4.3 ± 1.2
			1.5	6	37.8 ± 3.1	7.2 ± 6.5	4.7 ± 0.6
		KOH	0.75	12	53.6 ± 9.2	10.6 ± 9.2	5.8 ± 0.3
			1.5	6	56.9 ± 17.0	7.7 ± 9.0	4.6 ± 0.5
	3.2		0	18	60.0 ± 21.8	6.2 ± 5.5	5.6 ± 1.0
		NaOH	0.75	12	54.2 ± 2.3	6.7 ± 5.8	5.1 ± 0.6
			0.75	6	38.2 ± 2.7	8.7 ± 7.5	5.3 ± 0.3
		KOH	0.75	12	30.8 ± 2.9	13.8 ± 13.0	5.0 ± 1.6
			1.5	6	63.4 ± 35.0	10.3 ± 9.2	4.4 ± 0.5
Oat hull	1.6		0	12	36.7 ± 17.0	10.5 ± 9.1	7.5 ± 1.2
		NaOH	0.75	18	42.8 ± 11.3	15.6 ± 13.8	6.3 ± 1.0
			1.5	18	37.1 ± 8.5	14.3 ± 12 .6	4.2 ± 1.2
		KOH	1.5	18	56.4 ± 17.9	16.0 ± 13.8	4.8 ± 0.9
			1.5	6	41.8 ± 14.0	12.9 ± 11.5	5.7 ± 1.6
	3.2		0	6	51.2 ± 19.5	11.0 ± 9.6	9.2 ± 0.4
		NaOH	0.75	6	22.7 ± 11.0	12.9 ± 14.4	6.8 ± 2.2
			1.5	18	48.7 ± 8.3	14.4 ± 13.3	5.1 ± 0.8
		KOH	0.75	12	47.9 ± 18.2	16.0 ± 16.0	5.4 ± 0.6
			1.5	18	62.6 ± 2.0	10.4 ± 18.0	6.4 ± 1.3

C–cellulose; H–hemicellulose; L–lignin; ^a^ Mean ± standard deviation of three replicates.

**Table 6:** Effect of MW/alkali pre-treatments on pellet density, dimensional stability and tensile strength for canola straw pellets.

Sample	Screen Size (mm)	Alkali	Concentration (%)	Microwave Heating Time (min)	Pellets Density ^a^ (kg/m^3^)	Dimensional Stability ^a^ (%)	Tensile Strength ^b^ (MPa)
**Untreated canola straw**	1.6				1030.87 ± 9.89	3.95 ± 1.59	0.26 ± 0.09
	3.2				1060.82 ± 12. 99	5.23 ± 0.87	0.62 ± 0.27
**Canola straw**	1.6		0	6	1066.17 ± 28.38	0.51 ± 2.94	0.72 ± 0.31
			0	12	1037.10 ± 8.95	1.43 ± 1.25	0.74 ± 0.19
			0	18	1021.65 ± 7.92	3.27 ± 0.86	0.56 ± 0.15
		NaOH	0.75	6	1286.59 ± 14.34	0.05 ± 0.94	4.71 ± 0.74
			0.75	12	1309.35 ± 7.15	2.21 ± 0.52	2.66 ± 0.52
			0.75	18	1248.24 ± 9.12	1.13 ± 0.70	1.79 ± 0.28
			1.5	6	1319.21 ± 11.09	0.76 ± 0.91	5.22 ± 1.21
			1.5	12	1327.98 ± 6.72	0.78 ± 0.32	3.44 ± 1.02
			1.5	18	1370.27 ± 12.62	0.79 ± 0.62	2.31 ± 0.48
		KOH	0.75	6	1243.01 ± 8.53	0.04 ± 0.52	2.67 ± 0.41
			0.75	12	1195.28 ± 8.25	0.17 ± 0.68	1.90 ± 0.34
			0.75	18	1160.16 ± 8.15	1.44 ± 0.51	0.85 ± 0.30
			1.5	6	1392.21 ± 10.74	0.83 ± 0.58	3.78 ± 0.62
			1.5	12	1339.64 ± 7.42	0.26 ± 0.39	2.58 ± 0.37
			1.5	18	1321.01 ± 17.10	1.63 ± 2.51	2.11 ± 0.47
	3.2		0	6	1089.17 ± 19.24	0.16 ± 0.64	1.19 ± 0.44
			0	12	1086.86 ± 6.86	1.23 ± 0.69	1.04 ± 0.27
			0	18	1029.82 ± 6.51	3.98 ± 0.95	0.81 ± 0.40
		NaOH	0.75	6	1324.75 ± 9.68	2.60 ± 0.48	4.85 ± 0.99
			0.75	12	1283.60 ± 10.08	0.33 ± 0.47	2.53 ± 0.88
			0.75	18	1277.29 ± 12.02	1.85 ± 0.65	1.69 ± 0.42
			1.5	6	1351.61 ± 14.86	1.66 ± 0.36	4.20 ± 1.03
			1.5	12	1345.57 ± 8.66	0.17 ± 0.37	4.11 ± 1.53
			1.5	18	1388.30 ± 9.61	1.18 ± 0.44	2.59 ± 0.70
		KOH	0.75	6	1201.33 ± 5.62	0.70 ± 0.38	2.07 ± 0.55
			0.75	12	1220.50 ± 7.49	0.27 ± 0.62	1.73 ± 0.35
			0.75	18	1176.32 ± 8.63	2.10 ± 0.90	1.41 ± 0.39
			1.5	6	1382.62 ± 5.73	0.83 ± 0.53	5.19 ± 0.60
			1.5	12	1344.09 ± 8.06	0.29 ± 0.49	3.19 ± 0.90
			1.5	18	1355.93 ± 11.53	1.38 ± 0.77	2.89 ± 0.70

^a^ Mean ± standard deviation of ten pellets produced; ^b^ Mean ± standard deviation of thirteen specimens tables made.

**Table 7:** Effect of MW/Alkali pre-treatments on pellet density, dimensional stability and tensile strength for oat hull pellets.

Sample	Screen Size (mm)	Alkali	Concentration (%)	Microwave Heating Time (min)	Pellets Density ^a^ (kg/m^3^)	Dimensional Stability ^a^ (%)	Tensile Strength ^b^ (MPa)
**Untreated oat hull**	1.6				1031.23 ± 35.64	7.75 ± 3.26	0.04 ± 0.03
	3.2				1087.74 ± 13.16	6.14 ± 1.93	0.39 ± 0.27
**Oat hull**	1.6		0	6	989.14 ± 22.44	3.39 ± 1.48	0.14 ± 0.14
			0	12	1029.53 ± 12.57	9.34 ± 1.29	0.04 ± 0.02
			0	18	1028.72 ± 15.64	5.76 ± 6.05	0.30 ± 0.27
		NaOH	0.75	6	1238.12 ± 13.72	0.84 ± 0.42	1.34 ± 0.42
			0.75	12	1209.12 ± 13.71	1.38 ± 0.82	1.58 ± 0.75
			0.75	18	1221.99 ± 11.63	5.28 ± 1.08	1.33 ± 0.91
			1.5	6	1198.89 ± 16.53	0.53 ± 1.15	1.19 ± 0.64
			1.5	12	1286.52 ± 5.62	0.70 ± 0.49	1.96 ± 1.51
			1.5	18	1292.59 ± 30.61	4.54 ± 1.08	3.36 ± 1.63
		KOH	0.75	6	1123.85 ± 9.12	0.53 ± 0.67	0.57 ± 0.29
			0.75	12	1164.37 ± 9.76	0.34 ± 0.73	0.82 ± 0.44
			0.75	18	1166.59 ± 17.28	4.79 ± 1.22	0.73 ± 0.34
			1.5	6	1185.69 ± 24.27	1.04 ± 0.84	0.63 ± 0.31
			1.5	12	1220.42 ± 8.28	1.45 ± 0.65	0.83 ± 0.37
			1.5	18	1290.75 ± 18.82	3.30 ± 1.60	1.43 ± 0.65
	3.2		0	6	1045.82 ± 9.10	0.44 ± 0.58	0.25 ± 0.16
			0	12	1018.03 ± 23.14	4.67 ± 1.44	0.30 ± 0.26
			0	18	1066.38 ± 11.81	11.78 ± 1.88	0.45 ± 0.29
		NaOH	0.75	6	1205.73 ± 11.65	1.68 ± 0.58	1.23 ± 0.68
			0.75	12	1198.83 ± 7.30	1.82 ± 0.87	1.17 ± 0.71
			0.75	18	1219.29 ± 8.54	6.56 ± 0.86	1.91 ± 1.37
			1.5	6	1218.86 ± 32.75	1.16 ± 2.31	1.28 ± 0.54
			1.5	12	1321.34 ± 8.33	1.07 ± 0.97	2.27 ± 1.67
			1.5	18	1274.09 ± 13.01	6.15 ± 0.76	2.65 ± 1.18
		KOH	0.75	6	1073.31 ± 7.69	1.15 ± 0.75	0.46 ± 0.28
			0.75	12	1160.83 ± 8.34	6.22 ± 1.38	0.87 ± 0.95
			0.75	18	1143.75 ± 8.74	7.66 ± 1.12	0.90 ± 0.53
			1.5	6	1212.34 ± 6.39	0.83 ± 0.67	1.00 ± 0.64
			1.5	12	1248.13 ± 9.13	3.46 ± 0.69	1.08 ± 0.69
			1.5	18	1210.94 ± 21.26	5.95 ± 1.18	1.17 ± 0.76

^a^ Mean ± standard deviation of 10 pellets produced; ^b^ Mean ± standard deviation of 13 specimen tables made.

**Table 8:** ANOVA *p*-values showing the effect of alkali concentration and microwave heating time on pellet density, dimensional stability and tensile strength for canola straw.

**Sample**	**Screen Size (mm)**	**Alkali**	**Source of Variation**	**DF**	***p*-Values**		
					**Pellets’ Density (kg/m^3^)**	**Dimensional Stability (%)**	**Tensile Strength (MPa)**
Canola straw	1.6	NaOH	Model	5	0.005	0.138	0.020
			A—Alkali conc.	1	0.001	0.077	0.006
			B—Microwave time	1	0.641	0.096	0.018
			AB	1	0.153	0.556	0.076
			A^2^	1	0.014	0.192	0.093
			B^2^	1	0.753	0.128	0.511
			Residual	3			
			Cor Total	8			
			R-Squared		0.988	0.872	0.966
		KOH	Model	5	0.000	0.020	0.024
			A—Alkali conc.	1	<0.0001	0.040	0.005
			B—Microwave time	1	0.004	0.006	0.026
			AB	1	0.331	0.283	0.129
			A^2^	1	0.737	0.043	0.818
			B^2^	1	0.233	0.061	0.894
			Residual	3			
			Cor Total	8			
			R-Squared		0.998	0.966	0.962
**Sample**	**Screen Size (mm)**	**Alkali**	**Source of Variation**	**DF**	***p*-Values**		
					**Pellets’ Density (kg/m^3^)**	**Dimensional Stability (%)**	**Tensile Strength (MPa)**
Canola straw	3.2	NaOH	Model	5	0.003	0.070	0.132
			A—Alkali conc.	1	0.001	0.106	0.035
			B—Microwave time	1	0.289	0.013	0.094
			AB	1	0.120	0.569	0.531
			A^2^	1	0.015	0.793	0.338
			B^2^	1	0.780	0.703	0.994
			Residual	3			
			Cor Total	8			
			R-Squared		0.990	0.921	0.876
		KOH	Model	5	0.004	0.056	0.018
			A—Alkali conc.	1	0.001	0.052	0.004
			B—Microwave time	1	0.131	0.015	0.042
			AB	1	0.511	0.220	0.097
			A^2^	1	0.395	0.775	0.105
			B^2^	1	0.520	0.171	0.408
			Residual	3			
			Cor Total	8			
			R-Squared		0.989	0.932	0.970

DF = degree of freedom.

**Table 9:** ANOVA P-values showing the effect of alkali concentration and microwave heating time on pellet density, dimensional stability and tensile strength for oat hull.

**Sample**	**Screen Size (mm)**	**Alkali**	**Source of Variation**	**DF**	***p*-Values**		
					**Pellets’ Density (kg/m^3^)**	**Dimensional Stability (%)**	**Tensile Strength (MPa)**
Oat hull	1.6	NaOH	Model	5	0.019	0.352	0.054
			A—Alkali conc.	1	0.003	0.145	0.011
			B—Microwave time	1	0.262	0.179	0.119
			AB	1	0.492	0.699	0.105
			A^2^	1	0.040	0.327	0.475
			B^2^	1	0.621	0.833	0.805
			Residual	3			
			Cor Total	8			
			R-Squared		0.967	0.739	0.934
		KOH	Model	5	0.004	0.257	0.053
			A—Alkali conc.	1	0.001	0.096	0.011
			B—Microwave time	1	0.022	0.146	0.080
			AB	1	0.158	0.722	0.165
			A^2^	1	0.113	0.272	0.326
			B^2^	1	0.595	0.658	0.611
			Residual	3			
			Cor Total	8			
			R-Squared		0.989	0.796	0.936
**Sample**	**Screen Size (mm)**	**Alkali**	**Source of Variation**	**DF**	***p*-Values**		
					**Pellets’ Density (kg/m^3^)**	**Dimensional Stability (%)**	**Tensile Strength (MPa)**
Oat hull	3.2	NaOH	Model	5	0.041	0.002	0.017
			A—Alkali conc.	1	0.006	0.006	0.003
			B—Microwave time	1	0.434	0.000	0.034
			AB	1	0.697	0.024	0.103
			A^2^	1	0.175	0.038	0.246
			B^2^	1	0.805	0.088	0.784
			Residual	3			
			Cor Total	8			
			R-Squared		0.945	0.994	0.970
		KOH	Model	5	0.070	0.029	0.032
			A—Alkali conc.	1	0.010	0.116	0.005
			B—Microwave time	1	0.407	0.005	0.074
			AB	1	0.792	0.144	0.941
			A^2^	1	0.795	0.420	0.668
			B^2^	1	0.574	0.600	0.705
			Residual	3			
			Cor Total	8			
			R-Squared		0.922	0.957	0.955

DF = degree of freedom.

**Table 10:** Enzymatic saccharification for microwave-assisted, alkali pre-treated canola straw and oat hull.

Sample	Screen Size (mm)	Alkali	Concentration (%)	Microwave Heating Time (min)	Cellulose in Substrate ^a^ (%)	Average A540 Mean Value ^a^ (mg glc.)	Average Glucose ^a^ (mg/g)	Average Glucose Digestion Percentage ^a^ (%)
**Canola straw**	1.6		0	18	63.1 ± 32.0	0.574 ± 0.08	42.25 ± 5.99	6.70 ± 0.95
		NaOH	1.5	6	37.8 ± 3.1	0.128 ± 0.04	9.45 ± 2.93	2.50 ± 0.78
			1.5	18	59.1 ± 0.5	1.494 ± 0.12	110.05 ± 9.10	18.62 ± 1.54
		KOH	0.75	12	53.6 ± 9.2	0.338 ± 0.23	24.92 ± 16. 61	4.65 ± 3.10
			1.5	6	56.9 ± 17.0	0.725 ± 0.08	53.42 ± 6.07	9.39 ± 1.07
	3.2		0	18	60.0 ± 21.8	0.662 ± 0.16	48.75 ± 11.99	8.13 ± 2.00
		NaOH	0.75	6	38.2 ± 8.7	0.757 ± 0.14	55.78 ± 10.25	14.60 ± 2.68
			0.75	12	54.2 ± 2.3	0.482 ± 0.30	35.47 ± 22.37	6.54 ± 4.13
		KOH	0.75	12	30.8 ± 2.9	0.434 ± 0.08	31.96 ± 6.10	10.38 ± 1.98
			1.5	6	63.4 ± 35.0	1.314 ± 0.21	96.77 ± 15.31	15.26 ± 2.41
**Oat hull**	1.6		0	12	36.7 ± 17.0	0.086 ± 0.02	6.33 ± 1.36	1.73 ± 0.37
		NaOH	0.75	18	42.8 ± 11.8	1.346 ± 0.07	99.10 ± 4.79	23.16 ± 1.12
			1.5	18	37.1 ± 8.5	0.031 ± 0.01	2.26 ± 0.70	0.61 ± 0.19
		KOH	1.5	6	41.8 ± 14.0	1.324 ± 0.15	97.53 ± 11.32	23.33 ± 2.71
			1.5	18	56.4 ± 17.9	1.149 ± 0.38	84.64 ± 27.27	15.01 ± 4.96
	3.2		0	6	51.2 ± 19.5	0.073 ± 0.01	5.38 ± 0.51	1.05 ± 0.10
		NaOH	0.75	6	22.7 ± 11.0	0.981 ± 0.11	72.22 ± 7.98	31.82 ± 3.52
			1.5	18	48.7 ± 8.3	0.032 ± 0.01	2.38 ± 0.47	0.49 ± 0.10
		KOH	0.75	12	47.9 ± 18.2	0.452 ± 0.03	33.26 ± 2.51	6.94 ± 0.52
			1.5	18	62.6 ± 2.0	1.152 ± 0.29	84.87 ± 21.25	13.56 ± 3.39

^a^ Mean ± standard deviation of three replicates.

**Table 11:** Goal for optimization of variables during the experimental pelletization of canola straw and oat hull.

Variable	Goal	Level of Importance
Independent		
Alkali concentration (%)	In range (0 to 1.5)	
MW heating time (min)	In range (6 to 18)	
Dependent		
Pellet density (kg/m^−3^)	Maximize	3
Dimensional stability (%)	Minimize	2
Tensile strength (MPa)	Maximize	1

**Table 12:** Optimum conditions for producing canola straw and oat hull pellets under microwave-assisted alkali (NaOH and KOH) pre-treatment.

Sample	Screen Size (mm)	Alkali	Concentration (%)	Microwave Heating Time (min)	Pellets’ Density (kg/m^3^)	Dimensional Stability (%)	Tensile Strength (MPa)	Desirability
Canola straw	1.6	NaOH	1.28	6.93	1330.99	1.02	4.93	0.867
	3.2		1.48	6	1352.12	2.02	4.79	0.931
	3.2	KOH	1.5	6	1367.46	0.46	4.86	0.937
	1.6		1.5	6.28	1391.20	0.19	3.75	0.963
Oat hull	1.6	NaOH	1.38	14.72	1285.22	1.79	2.41	0.789
	3.2		1.5	13.66	1282.49	2.36	2.20	0.796
	1.6	KOH	1.49	18	1276.29	3.40	1.33	0.797
	3.2		1.5	9.01	1226.16	1.66	1.03	0.851

## References

[1] Nomanbhay S.M., Hussain R., Palanisamy K. (2013). Microwave-assisted alkaline pretreatment and microwave assisted enzymatic saccharification of oil palm empty fruit bunch fiber for enhance fermentable sugar yield. J. Sustain. Bioenergy Syst..

[2] Alvira P., Tomas-Pejo E., Ballesteros M., Negro M.J. (2010). Pretreatment technologies for an efficient bioethanol production process based on enzymatic hydrolysis: A review. Bioresour. Technol..

[3] Balat M., Balat H., Oz C. (2008). Progress in bioethanol processing. Prog. Energy Combust. Sci..

[4] Smith D. (2013). Brief Overview of Biofuels and Introduction to Feedstock.

[5] Liu T., McConkey B., Huffman T., Smith S., MacGregor B., Yemshanov D., Kulshreshtha S. (2014). Potential and impacts of renewable energy production from agricultural biomass in Canada. Appl. Energy.

[6] Demirbas A., Balat M.F.M., Balat H. (2009). Potential contribution of biomass to the sustainable energy development. Energy Convers. Manag..

[7] Ohgren K., Bura R., Lesnicki G., Saddler J., Zacchi G. (2007). A comparison between simultaneous saccharification and fermentation using steam-pretreated corn stover. Process Biochem..

[8] Sorda G., Banse M., Kemfert C. (2012). An overview of biofuel policies across the world. Energy Policy.

[9] Mosier N.S., Wyman C., Dale B., Elander R., Lee Y.Y., Holtzapple M., Ladisch M.R. (2005). Features of promising technologies for pretreatment of lignocellulosic biomass. Bioresour. Technol..

[10] Sanchez O.J., Cardona C.A. (2008). Trends in biotechnological production of fuel ethanol from different feedstocks. Bioresur. Technol..

[11] Li X., Mupondwa E., Panigraphi S., Tabil L.G., Sokhansanj S., Stumborg M. (2012). A review of agricultural crop residue supply in Canada for Cellulosic ethanol production. Renew. Sustain. Energy Rev..

[12] Saskatchewan Ministry of Agriculture Field Crop Sheet 2014. Field Crop Statistics. www.publications.gov.sk.ca/redirect.cfm?p=74877&i=83776.

[13] Agbor V.B., Cicek N., Sparling R., Berlin A., Levin B.D. (2011). Biomass pretreatment: Fundamentals toward application. Biotechnol. Adv..

[14] Quintero J.A., Rincon L.E., Cardona C.A., Pandey A., Larroche C., Ricke S.C., Dussap C.G., Gnansounou E. (2011). Production of bioethanol from agro industrial residues as feedstocks. Biofuels: Alternative Feedstocks and Conversion Processes.

[15] Zhu S., Wu Y., Yu Z., Zhang X., Wang C., Yu F., Jin S. (2006). Production of ethanol from microwave-assisted alkali pretreated wheat straw. Process Biochem..

[16] Zhao Y., Wang Y., Zhu J.Y., Ragauskas A., Deng Y. (2008). Enhanced enzymatic hydrolysis of spruce by alkaline pretreatment at low temperature. Biotechnol. Bioeng..

[17] Ooshima H., Aso K., Harano Y., Yamamoto T. (1984). Microwave treatment of cellulosic materials for their enzymatic hydrolysis. Biotechnol. Lett..

[18] Azuma J.I., Tanaka F., Koshijima T. (1984). Enhancement of enzymatic susceptibility of lignocellulosic wastes by microwave irradiation. J. Ferment. Technol..

[19] Gong G., Liu D., Huang Y. (2010). Microwave-assited organic acid pretreatment for enzymatic hydrolysis of rice straw. Biosyst. Eng..

[20] Quitain A.T., Sasaki M., Goto M., Fang Z. (2013). Microwave-based pretreatment for efficient biomass-to-biofuel conversion. Pretreatment Techniques for Biofuels and Biorefineries.

[21] Keshwani D.R., Cheng J.J. (2010). Microwave-based alkali pretreatment of switchgrass and coastal bermudagrass for bioethanol production. Biotechnol. Prog..

[22] Sun Y., Cheng J. (2002). Hydrolysis of lignocellulosic materials for ethanol production: A review. Bioresour. Technol..

[23] Kashaninejad M., Tabil L.G. (2011). Effect of microwave: chemical pretreatment on compression characteristics of biomass grinds. Biosyst. Eng..

[24] Xu J., Pandey A., Negi S., Binod P. (2015). Microwave pretreatment. Pretreatment of Biomass: Processes and Technologies.

[25] Rodrigues T.H.S., Rocha M.V.P., Macedo G.R.D., Goncalves L.R.B. (2011). Ethanol production from cashew apple bagasse: Improvement of enzymatic hydrolysis by microwave-assisted alkali pretreatment. Appl. Biochem. Biotechnol..

[26] Saha B.C., Biswas A., Cotta M.A. (2008). Microwave pretreatment, enzymatic saccharification and fermentation of wheat straw to ethanol. J. Biobased Mater. Bioenergy.

[27] Binod P., Satyanagalakshmi K., Sindhu R., Janu K.U., Sukumaran R.K., Pandey A. (2012). Short duration microwave assisted pretreatment enhances the enzymatic saccharification and fermentable sugar yield from sugarcane bagasse. Renew. Energy.

[28] Choudhary R., Umagiliyage A.L., Liang Y., Siddaramu T., Haddock J., Markevicus G. (2012). Microwave pretreatment for enzymatic saccharification of sweet sorghum bagasse. Biomass Bioenergy.

[29] Ethaib S., Omar R., Kamal S.M.M., Biak D.R.A. (2015). Microwave-assisted pretreatment of lignocellulosic biomass: A review. J. Eng. Sci. Technol..

[30] Taherzadeh M.J., Karimi K. (2008). Pretreatment of lignocellulosic waste to improve ethanol and biogas production: a review. Int. J. Mol. Sci..

[31] Sindhu R., Pandey A., Binod P., Pandey A., Negi S., Binod P., Larroche C. (2015). Alkaline treatment. Pretreatment of Biomass: Process and Technologies.

[32] Sharma R., Palled V., Sharma-Shivappa R.R., Osborne J. (2013). Potential of potassium hydroxide pretreatment of switch grass for fermentable sugar production. Appl. Biochem. Biotechnol..

[33] Adapa P.K., Tabil L.G., Schoenau G. (2013). Factors affecting the quality of biomass pellet for biofuel and energy analysis of pelleting process. Int. J. Agric. Biol. Eng..

[34] Veal M.W., Cheng J. (2010). Biomass logistics. Biomass to Renewable Energy Processes.

[35] Mani S., Tabil L.G., Sokhansanj S. (2006). Effects of compressive force, particle size and moisture content on mechanical properties of biomass pellets from grasses. Biomass Bioenergy.

[36] Jenkins B.M., Baxter L.L., Koppejan J., Brown R.C. (2011). Biomass combustion. Thermochemical Processing of Biomass, Conversions into Fuels, Chemical and Power.

[37] Adapa P., Tabil L.G., Schoenau G. (2011). Grinding performance and physical properties of non-treated and steam exploded barley, canola, oat and wheat straw. Biomass Bioenergy.

[38] ASABE (2006). ASABE Standard S358.2. Moisture measurement–forages. ASABE Standards.

[39] Adapa P., Tabil L.G., Schoenau G. (2009). Compaction characteristics of barley, canola, oat and wheat straw. Biosyst. Eng..

[40] ASABE (2008). ASABE Standard S319.4. Method of determining and expressing fineness of feed materials by sieve. ASABE Standards.

[41] Iroba K.L., Tabil L.G., Zhang B., Wan Y. (2013). Lignocellulosic biomass feedstock characteristics, pretreatment methods and pre-processing of biofuel and bioproduct applications. Biomass Processing, Conversion and Biorefinery.

[42] Sluiter A., Hames B., Ruiz R., Scarlata C., Sluiter J., Templeton D., Crocker D. (2007). Determination of Structural Carbohydrates and Lignin in Biomass.

[43] Sluiter A., Hames B., Ruiz R., Scarlata C., Sluiter J., Templeton D., Crocker D. (2008). Determination of Ash in Biomass.

[44] Tabil L.G., Sokhansanj S. (1997). Bulk properties of alfalfa grind in relation to its compaction characteristics. Appl. Eng. Agric..

[45] Kashaninejad M.B., Kashaninejad B., Tabil L.G. (2010). Effect of Microwave Pretreatment on Densification of Wheat Straw.

[46] Fell J.T., Newton J.M. (1968). The tensile strength of lactose tablets. J. Pharm. Pharmacol..

[47] Fell J.T., Newton J.M. (1970). Determination of tablet strength by the diametral-compression test. J. Pharm. Sci..

[48] Ryu D.D., Mandels M. (1980). Cellulases-biosynthesis and applications. Enyzme Microb. Technol..

[49] Xiao Z., Stroms R., Tsang A. (2004). Microplate-based filter paper to measure total cellulase activity. Biotechnol. Bioeng..

[50] Wood T.M., Bhat K.M., Willis A.W., Scott T.K. (1988). Methods for measuring cellulase activities. Methods Enzymol..

[51] Lan T.Q., Lou H., Zhu J.Y. (2013). Enzymatic saccharification of lignocelluloses should be conducted at elevated pH 5.2–6.2. Bioenergy Res..

[52] Diaz A.B., Moretti M.M., Bezerra-Bussoli C., Nunes C.D., Blandino A., da Silva R., Gomes E. (2015). Evaluation of microwave-assisted pretreatment of lignocellulosic biomass immersed in alkaline glycerol for fermentable sugars. Bioresour. Technol..

[53] Oguocha I. (2015). Materials Characterization Techniques.

[54] Yue Z.B., Yu H.Q., Hu Z.H., Harada H., Li Y.Y. (2008). Surfactant-enhanced anaerobic acidogenesis of *Canna indica* L. by rumen cultures. Bioresour. Technol..

[55] Ma H., Liu W., Chen X., Wu Y., Yu Z. (2009). Enhanced enzymatic saccharification of rice straw by microwave pretreatment. Bioresour. Technol..

[56] Kumar P., Barrett D.M., Delwiche M.J., Stroeve P. (2009). Methods for pretreatment of lignocellulosic biomass for efficient hydrolysis and biofuel production. Ind. Eng. Chem. Res..

[57] Zhu S., Wu Y., Yu Z., Liao Z.J., Zhang Y. (2005). Pretreatment by microwave/alkali of rice straw and its enzymatic hydrolysis. Process Biochem..

[58] Zhu S., Wu Y., Yu Z., Chen Q., Wu G., Yu F., Wang C., Jin S. (2006). Microwave-assisted alkali pre-treatment of wheat straw and its enzymatic hydrolysis. Biosyst. Eng..

[59] Tomas-Pejo E., Alvria P., Ballesteros M., Negro M.J., Pandey A., Larroche C., Ricke S.C., Dussap C.G., Gnansounou E. (2011). Pretreatment technologies for lignocellulose to bioethanol conversion. Biofuels: Alternative Feedstocks and Conversion Processes.

[60] Iroba K.L., Tabil L.G., Sokhansanj S., Meda V. (2014). Producing durable pellets from barley straw subjected to radio frequency-alkaline and steam explosion pretreatments. Int. J. Agric. Biol. Eng..

[61] Tabil L.G. (1996). Binding and Pelleting Characteristics of Alfalfa. Ph.D. Thesis.

[62] Anna C.S., de Souza W. (2012). Microscopy as a Tool to Follow Deconstruction of Lignocellulosic Biomass. www.formatex.info/microscopy5/book/639-645.pdf.

